# Invasive *Haemophilus influenzae* disease in the vaccine era in Rio de Janeiro, Brazil

**DOI:** 10.1590/0074-02760160391

**Published:** 2017-02-16

**Authors:** Mari Tuyama, Jessica Corrêa-Antônio, Jessica Schlackman, Jane W Marsh, Maria C Rebelo, Elaine O Cerqueira, Márcio Nehab, Fabíola Kegele, Getúlio F Carmo, Dominique CA Thielmann, Paulo F Barroso, Lee H Harrison, David E Barroso

**Affiliations:** 1 Fundação Oswaldo Cruz-Fiocruz, Instituto Oswaldo Cruz, Laboratório de Epidemiologia e Sistemática Molecular, Rio de Janeiro, RJ, Brasil; 2 University of Pittsburgh, School of Medicine and Graduate School of Public Health, Infectious Diseases Epidemiology Research Unit, Pittsburgh, PA, United States of America; 3 Secretaria de Estado de Saúde do Rio de Janeiro, Assessoria de Meningites, Rio de Janeiro, RJ, Brasil; 4 Fundação Oswaldo Cruz-Fiocruz, Instituto Fernandes Figueira, Rio de Janeiro, RJ, Brasil; 5 CientíficaLab, Rio de Janeiro, RJ, Brasil; 6 Universidade Federal do Rio de Janeiro, Faculdade de Medicina, Rio de Janeiro, RJ, Brasil

**Keywords:** Haemophilus influenzae, meningitis, MLST, vaccine, serotypes

## Abstract

**BACKGROUND:**

*Haemophilus influenzae* (Hi) serotype b (Hib) conjugate vaccine was incorporated into the infant immunisation schedule in Brazil in 1999, where Hib was one of the major etiologic sources of community-acquired bacterial meningitis.

**OBJECTIVES:**

The purpose of this study is to describe the molecular epidemiology of invasive Hi disease in Rio de Janeiro state, Brazil, before and after vaccine introduction.

**METHODS:**

Surveillance data from 1986 to 2014 were analysed. Hi isolates recovered from cerebrospinal fluid (CSF) or blood from 1993 to 2014 were serotyped by slide agglutination, genotyped by multilocus sequence typing (MLST), and the capsule type evaluation, differentiation of serologically non-typeable isolates, and characterisation of the capsule (*cap*) locus was done by polymerase chain reaction. Antimicrobial susceptibility testing was performed using E-test.

**FINDINGS:**

From 1986 to 1999 and from 2000 to 2014, 2580 and 197 (42% without serotype information) confirmed cases were reported, respectively. The case fatality rate was 17% and did not correlate with the strain. Hib and b^-^ variant isolates belonged to ST-6, whereas serotype a isolates belonged to the ST-23 clonal complex. Serotype a appeared to emerge during the 2000s. Non-encapsulated isolates were non-clonal and distinct from the encapsulated isolates. Ampicillin-resistant isolates were either of serotype b or were non-encapsulated, and all of them were β-lactamase-positive but amoxicillin-clavulanic acid susceptible.

**MAIN CONCLUSIONS:**

Although Hi meningitis became a relatively rare disease in Rio de Janeiro after the introduction of the Hib conjugate vaccine, the isolates recovered from patients have become more diverse. These results indicate the need to implement an enhanced surveillance system to continue monitoring the impact of the Hib conjugate vaccine.

*Haemophilus influenzae* (Hi) serotype b (Hib) infection is endemic globally and produces a large spectrum of clinical manifestations. Meningitis is the most common; however, the disease spectrum includes pneumonia, epiglottitis, septicaemia, cellulitis, and septic arthritis (Peltola 2000). Children between three months and three years of age have the highest disease incidence, with 95% of meningitis cases occurring in children aged less than five years (Peltola 2000, Kelly et al. 2004). Records on Hi meningitis maintained in Rio de Janeiro state, Brazil, date back to 1986. The introduction of the Hib conjugate vaccine (PRP-T) for routine use in infants as a part of the national immunisation program in Rio de Janeiro occurred in July 1999. Since then, high vaccine coverage in children aged less than one year has been achieved and a sharp reduction in reported cases has been observed (Ribeiro et al. 2007, Zanella et al. 2011). In Rio de Janeiro, the average vaccination coverage is 96%, and the combined vaccine DTP-Hib, which replaced PRP-T vaccine in 2002, is used.

The Hi capsular polysaccharide is a cell-surface structure comprised of long carbohydrate chains that play an important role in determining bacterial virulence and are used to evade host immune defences (Kelly et al. 2004). In an animal model, Hi expressing capsular polysaccharide b has been shown to be the most virulent, followed by serotype a (Zwahlen et al. 1989). The capsule locus (*cap*) of all six Hi capsular types has a common organisation consisting of three functional regions (Leaves et al. 1995). Region I (ATP-driven export) contains four genes, i.e., *bexA-D*, involved in export of the polysaccharide to the cell surface (Kroll et al. 1990). Region III (post-polymerisation) contains the genes, *hcsA* and *hcsB*, associated with post-translational modifications and expression of the capsule (Sukupolvi-Petty et al. 2006). Region II (serotype-specific), located between these two functional regions, encodes the genes (*a-fcs1-4*) associated with biosynthesis of serotype-specific carbohydrates and are unique to each of the six capsular types (van Eldere et al. 1995, Satola et al. 2003).

In a typical Hib strain, regions I and III are flanked by the insertion element IS*1016*, which exists in duplicate with the presence of an IS*1016-bexA* partial deletion in one of the duplicate copies (Satola et al. 2003). This deletion stabilises duplication, prevents loss of encapsulation, and results in increased capsular polysaccharide production, which together are likely to contribute towards virulence (Gilsdorf et al. 2004). This mutation may also be associated with virulence in serotypes other than serotype b, since this *cap* locus organisation has also been found in other serotypes (Ogilvie et al. 2001).

The typical duplicated *cap* locus in Hib can undergo a recombination event resulting in loss of the intact *bexA* copy; the remaining truncated copy containing the *bexA* deletion results in the loss of capsule expression despite the presence of an intact region II (Leaves et al. 1995). This recombination event results in capsule-deficient mutants of Hib (b^-^) that retain all other attributes of the encapsulated form (Satola et al. 2003). These b^-^ variants can be differentiated by polymerase chain reaction (PCR)-based methods (Falla et al. 1994, Leaves et al. 1995, Satola et al. 2003).

We present here invasive Hi disease surveillance data, and results focused on the serotype b conjugate vaccine era of prevalence of different serotypes and genetic diversity of invasive isolates in Rio de Janeiro state.

## MATERIALS AND METHODS

*Surveillance data* - Public health surveillance of Hi disease in Rio de Janeiro state was conducted by the Meningitis Advisory Committee of the State Department of Health. Only reporting of Hi meningitis is mandatory and is based on isolation of Hi from a normally sterile site or detection by culture-independent diagnostic tests (antigen detection or PCR). There was no significant change in the surveillance system in the vaccine era. The results obtained with PCR to detect *H. influenzae* are restricted to the period of validation of a PCR assay between 2004 and 2006; PCR has not been introduced into routine practice (Tuyama et al. 2008). From 1986 to 2014, surveillance data were provided by the State Department of Health. Between 1986 and 1992, only the total number of new cases was reported; no information on serotype was available. Moreover, the vaccination status of patients is unknown. The population of Rio de Janeiro was 14,391,282 in 2000 and 15,989,929 in 2010. Clinical and epidemiological data were analysed using EpiInfo^TM^ (version 3.5.4, Centers for Disease Control and Prevention, Atlanta, GA, USA). The heterogeneity of proportions between groups was compared using the chi-squared test with Yates’s correction for statistical significance.

*Collection, serotype identification, and antimicrobial susceptibility testing of clinical isolates* - Heated blood agar was used to culture bacteria from the cerebrospinal fluid (CSF) or blood culture of patients with clinically diagnosed meningitis or other clinical manifestations. From 2000 to 2014, 65 stored Hi isolates recovered from invasive disease (meningitis = 46, septicaemia = 14, pneumonia = 5) were included. In addition, 72 (12%) isolates randomly selected from a list of 580 stored Hi samples, all characterised as serotype b and recovered from patients with meningitis between 1993 and 1999, were studied. Bacterial growth on Haemophilus test medium (HTM) agar plates (Oxoid, Basingstoke, Hampshire, UK) containing HTM supplement with the appearance of Gram-negative coccobacillus were identified using api NH (bioMérieux, Marcy-l’Étoile, France), which include a test for β-lactamase production. The serotype was determined by slide agglutination with specific rabbit antisera (BD Difco, Sparks, MD, USA), according to the manufacturer’s instructions.

Minimal inhibitory concentrations (MICs) of ampicillin, amoxicillin-clavulanic acid, ceftriaxone, rifampicin, chloramphenicol, cotrimoxazole, and azithromycin were determined using the E-test (bioMérieux, Marcy-l’Étoile, France), according to the manufacturer’s instructions. Briefly, a single E-test strip was placed onto HTM agar plates, which were incubated at 35ºC in a 5% CO_2_/95% air atmosphere for 20-24 hours. MIC interpretative standards for Hi were based on the Clinical and Laboratory Standards Institute document M100-S20 (CLSI 2010).

*PCR-based capsular typing and characterisation of the cap loci* - One isolated colony of each isolate was subcultured on an HTM agar plate, and DNA was isolated from the resuspended bacteria after overnight culture, using a QIAamp DNA Mini Kit (Qiagen, Hilden, Germany), according to the manufacturer’s protocol for DNA purification for Gram-negative bacteria.

PCR-based capsular typing was performed using a set of primers described previously – HI-I/HI-II (annealing to intact copy of *bexA*) and a-fI/a-fII (annealing to the central type-specific region II) (van Ketel et al. 1990, Falla et al. 1994). The first set of primer tests for the presence of *bexA*, which determines whether an isolate is encapsulated or non-encapsulated (van Ketel et al. 1990). The second step is used to determine each of the six specific capsule types (Falla et al. 1994).

We used three primers in two combinations – i.e., ORF6/BexB (3′ end junction-*bexB*) and ISLOUT/BexB (IS*1016-bexB*) – to identify the capsule polysaccharide synthesis gene structure (Leaves et al. 1995). This PCR combination (i) detects a *cap* locus consisting of multiple copies of *cap*; (ii) differentiates b^-^ from Hib isolates with a positive amplification reaction specific to b^-^ variants; (iii) identifies intact tandemly repeated copies of intact *cap* with no deletion in *bexA*; and (iv) identifies the partial deletion of *bexA* and IS*1016* in at least one copy of *cap*.

*Genotyping* - Multilocus sequence typing (MLST) was performed as previously described and used to determine genetic lineage (Meats et al. 2003). Allele and sequence type (ST) numbers were assigned by submission to the Hi MLST database (http://pubmlst.org/hinfluenzae/). MLST data were analysed against all STs found in the online database to organise the population into clonal complexes, which is defined as a group of STs in a population that shares four to seven alleles with at least one other ST in the group (Meats et al. 2003). Bionumerics software (version 5.10; Applied Maths) was used to create minimum spanning trees (Feil et al. 2004). We correlated *pgi* alleles with the expression of the capsule to test the accuracy of *pgi* genotyping as a surrogate for capsule typing (Anyanwu et al. 2003). The *pgi* allele sequences were compared with those alleles as reported on pubmlst.org/hinfluenzae.

*Ethical considerations* - The study was approved (CAAE 33354114.1.0000.5248) by the Ethical Committee of the Oswaldo Cruz Institute (CEP FIOCRUZ/IOC), Brazilian Ministry of Health.

## RESULTS

**Fig. 1:**
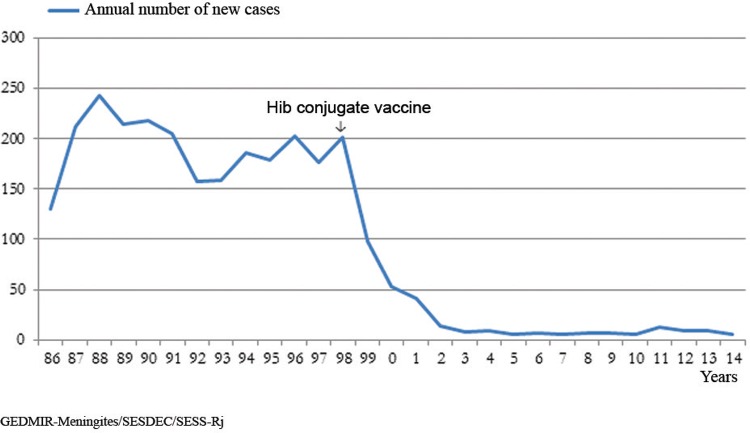
: reported number of *Haemophilus influenzae* meningitis cases in Rio de Janeiro state, Brazil, 1986-2014.

*Surveillance data* - A dramatic decline in the number of Hi meningitis cases occurred following introduction of conjugate vaccine in 1999 (Fig. 1). From 1986 to 1999 and from 2000 to 2014, 2580 and 197 confirmed cases were reported, representing an average of 184 and 13 cases per year, respectively. From 1993 to 1999, 1206 reported meningitis cases (172 cases per year) were attributed to serotype b and were confirmed by culture (720; CSF = 691; blood = 29) or antigen detection (486). Subsequently, from 2000 to 2014, 197 meningitis cases were confirmed by culture (79; CSF = 54; blood = 25), antigen detection (106), or PCR (12); among them, 11 (6%) were serotype a, 103 (52%) were serotype b, and 83 (42%) were without serotype information.

The time periods with the serotypes, the age category, the clinical manifestations (meningitis and non-meningitis cases), and the case fatality rates (CFR) are presented in Table I. Most serotype b isolates were recovered from children aged five years and younger; however, a reduction in the proportion of cases in this age category was noticed in the vaccine era. From the year 2000, patients with haemorrhagic rash (10%; 19/197) were children under 10 years of age (70%; < 1-year-old) and were more frequently infected with serotype a. The CFR was similar for both periods, but a marked increase was observed after 2010.

**TABLE I:** The time periods with the serotypes, the age category, the clinical manifestations, and the case fatality rates of *Haemophilus influenzae* disease in Rio de Janeiro state, Brazil, 1993-2014

Time period Serotype (number of patients)	Age category	Meningitis	Septicaemia	Pneumonia	Petechial rash	Case fatality rates
1993 - 1999 (1206)						17.5%^c^ (212/1206)
Serotype b (1203)	92%^*a*^ < 5-years	100%	-	-	-	17.6% (212/1203)
b- variant (2)	1-year and 14-years	100%	-	-	-	0%
Unencapsulated (1)	1-year	100%	-	-	-	0%
2000 - 2014 (220)						15.4%^c^ (30/194^d^)
Serotype a (12)	92% < 5-years	92%	Single patient	-	42%^b^	25% (25% 2000-2009)
Serotype b (104)	78%^*a*^ < 5-years	98%	Single patient	Single patient	9%^b^	25% (6% 2000-2009)
b- variant (1)	2-years	100%	-	-	-	0%
Unencapsulated (20)	70% < 5-years	20%	60%	20%	-	33% (0% 2000-2009^e^)
Without serotype information (83)	64% < 5-years	100%	-	-	6%	38% (18% 2000-2009)

*a*: (χ^2^ = 46; *p* < 0.01); *b*: (χ^2^ = 8.2; *p* < 0.01); *c*: (*p* = 0.38); *d*: three missing values; *e*: total of two patients in the period.

*PCR-based capsular typing* - Using PCR, 113 typeable isolates yielded a 343-bp amplicon when primers HI-I/HI-II were used, whereas no product was amplified from 24 non-typeable isolates. Of the 72 isolates included from 1993 to 1999 originally characterised at the time of isolation as serotype b, two were determined to be b^-^ variants and one was determined as non-encapsulated by PCR capsule typing. Of the 65 isolates recovered from the year 2000 and initially reported to be serotype b (n = 35), one was a b^-^ variant and two were non-encapsulated. Thus, in the bacterial collection of 137 viable Hi isolates recovered from patients with meningitis (n = 118), septicaemia (n = 14), or pneumonia (n = 5) (Table I) were found, out of which 12 were serotype a (first isolate found in 2006), 101 serotype b (1993-1999 = 69; 2000-2014 = 32), three were b^-^ variants (first isolate found in 1994), and 21 were non-encapsulated (first isolate found in 1993).

*Characterisation of the cap loci* - Hib isolates yielded a 3-kb product, which is an indication of a *cap* locus consisting of tandem repeat copies of *cap*, when ORF6/BexB primers were used. The ISLOUT/BexB primers produced two amplification products – a 300-bp amplicon, indicating the presence of partial deletion of IS*1016-bexA*, and a 1.5-kb amplicon, resultant of amplification across intact *bexA*. These results are consistent with a *cap* locus structure formed by multiple copies of *cap* and at least one copy of *cap* with an IS*1016-bexA* partial deletion. The b^-^ variants were genotypically serotype b; no product was amplified with primers ORF6/BexB and only the 300-bp amplicon was amplified with primers ISLOUT/BexB indicating the absence of intact *bexA*. PCR-based methods showed intact tandem-repeated copies of the *cap* locus with no deletion of *bexA* in Hi serotype a (Hia) isolates. The ORF6/BexB primers amplified a 3-kb product, whereas primers ISLOUT/BexB yielded only a 1.5-kb product. The PCR results of non-encapsulated isolates showed no amplification with these primers.

**Fig. 2:**
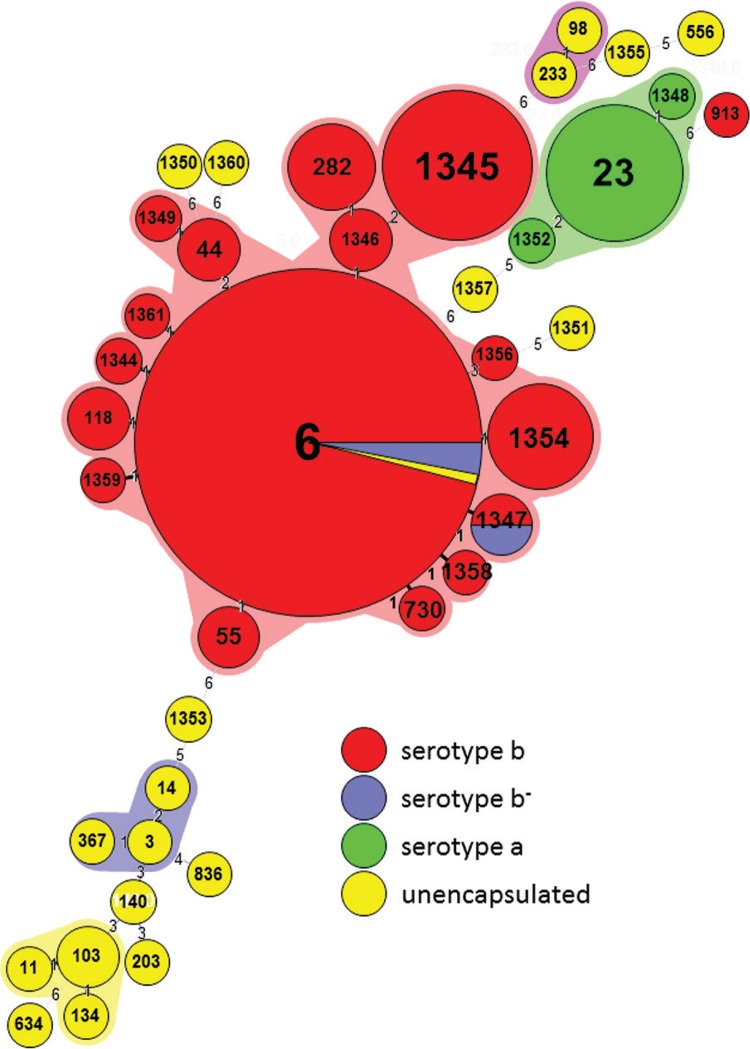
: *Haemophilus influenzae* clustered by multilocus sequence typing clonal complex, labelled by sequence type, colour coded by serotype.

*Genotyping* - By MLST, Hib (61%; 62/101) and b^-^ (67%; 2/3) isolates were ST-6 or a ST of the same clonal complex (four to seven identical alleles), of which 10 (67%; 10/15) were novel (Fig. 2), and a single isolate, ST-913, which is genetically distant to the ST-6 clonal complex. *H. influenzae* serotype a isolates were ST-23 (83%; 10/12) or two new STs closely related to the ST-23 clonal complex (Fig. 2) – ST-1348 (a single-locus variant of ST-23 that differs at *frdB*) and ST-1352 (a double-locus variant of ST-23 that differs at *frdB* and *recA*). Non-encapsulated isolates were non-clonal and distinct from the encapsulated isolates, with the exception of a single isolate of the ST-6 (Fig. 2). New STs were present across both periods of the study – before and after the vaccine introduction.

Capsule-associated *pgi* alleles were found among encapsulated isolates. All Hia isolates shared a single *pgi* allele (allele 11), which is conserved within the ST-23 serotype a isolates. Mostly Hib had *pgi* allele 7 (82%) or other alleles previously reported for this serotype (alleles: 3, 29, 31, and 89); b^-^ variant isolates also had *pgi* allele 7. Non-encapsulated Hi isolates had *pgi* alleles associated primarily with non-encapsulated isolates, of which one was novel (allele 226; ST-1357). However, one isolate (ST-6) had a *pgi* allele 7 and another (ST-1351) had a *pgi* allele 28, which are associated primarily with serotype b and e, respectively.

*Antimicrobial susceptibility testing* - All isolates were susceptible to ceftriaxone (MICs, 0.002-0.016 µg/mL), rifampicin (MICs, 0.006-0.75 µg/mL), azithromycin (MICs, 0.032-4 µg/mL), and amoxicillin-clavulanic acid (MICs, 0.125-1 µg/mL). The results of antimicrobial susceptibility testing of resistant isolates are shown in Table II. Resistance was detected for ampicillin (MICs, 4- > 256 µg/mL), cotrimoxazole (MICs, 1- > 32 µg/mL), and chloramphenicol (MICs, 8-16 µg/mL). Notably, all chloramphenicol-resistant isolates were recovered before the year 2002.

**TABLE II:** Results of the antimicrobial susceptibility testing of *Haemophilus influenzae*-resistant isolates recovered from patients in Rio de Janeiro state, Brazil, 1993-2014

Time period Serotype (number of isolates)	β-lactamase-positive	Ampicillin MIC ≥ 4 µg/mL	Chloramphenicol MIC ≥ 8 µg/mL	Ampicillin* and chloramphenicol	Cotrimoxazole MIC ≥ 1 µg/mL
1993 - 1999 (72)					
Serotype b (69)	12% (8/69)	12% (8/69)	14% (10/69)	12% (8/69)	10% (7/69)
b- variant (2)	0%	0%	0%	0%	0%
Unencapsulated (1)	0%	0%	0%	0%	0%
2000 - 2014 (65)					
Serotype a (12)	0%	0%	0%	0%	8% (1/12)
Serotype b (32)	16% (5/32)	16% (5/32)	9% (3/32)	9% (3/32)	3% (1/32)
b- variant (1)	0%	0%	Single isolate	0%	0%
Unencapsulated (20)	25% (5/20)	25% (5/20)	5% (1/20)	5% (1/20)	30% (6/20)

MIC: minimal inhibitory concentration; *: ampicilin MIC ≥ 4 µg/mL and chloranphenicol MIC ≥ 8 µg/mL.

## DISCUSSION

Hib conjugate vaccine has led to a marked and sustained reduction in the incidence of serotype b infection wherever it was introduced (Peltola 2000). The serotype-specific nature of the vaccine and the reduction in carriage of serotype b has raised concerns about the possibility of capsule replacement following introduction of vaccine. However, sustained increase in the invasive disease caused by non-serotype b strains has not been observed (Ladhani et al. 2008). A few reports have shown that such an increase does not correlate with meningitis cases, e.g., isolates are recovered from older adults, which has led to a change in the epidemiological pattern of invasive Hi disease. In Brazil, the same scenario has been reported following the widespread use of the conjugate vaccine in infancy since 1999; there is no evidence suggesting capsule serotype replacement or increase in meningitis due to non-encapsulated Hi (Lima et al. 2010, Zanella et al. 2011). Nevertheless, a temporary increase in non-b serotypes was observed in north-east Brazil (Ribeiro et al. 2007) and an increase in meningitis cases caused by non-encapsulated Hi was also reported (Zanella et al. 2011).

Regardless, the reduction of Hib disease has led to the occurrence of a higher proportion of cases attributable to non-serotype b or non-encapsulated isolates. These events, together with the reduction of incidence in children, may cause an upward shift in the age distribution, as a consequence of the tendency of non-encapsulated and some encapsulated isolates other than serotype a to affect the older age groups (Livorsi et al. 2012).

Although Hi became a relatively rare cause of community-acquired invasive bacterial infection in Rio de Janeiro after the introduction of the Hib conjugate vaccine, the isolates recovered from patients were more diverse, with the emergence of serotype a isolates during 2000s. A limitation of our study is that a substantial proportion of isolates not tested lack serotype information in the vaccine era. Improvement in surveillance could include implementation of more sensitive laboratory methods, such as, PCR-based methods (Satola et al. 2007), for more accurate estimates of the disease burden. Additionally, the reporting could be expanded to all the patients with invasive diseases, not just those with Hi meningitis. In addition, it is likely that our surveillance system substantially underestimated the true burden of Hi infection in our population.

The proportion of isolates characterised was low compared with the total number of cases reported, in particular before the introduction of the conjugate vaccine. Nevertheless, the molecular typing results are likely to be a representative of meningitis cases caused by Hi in Rio de Janeiro, taking into account the clonal nature of this species and the low frequency of serotypes other than serotypes a or b in Brazil (Zanella et al. 2011).

We present here for the first time the results of genotyping and *cap* locus characterisation of a collection of Hi isolates from Brazil, which revealed a genetically diverse population of encapsulated and non-encapsulated isolates causing invasive disease after the introduction of the conjugate vaccine. We demonstrated that Hia and Hib isolates belonged to two distinct clonal complexes, cc23 and cc6, respectively. In both clonal complexes, new STs were identified, which is consistent with an evolved population. ST-23 lineage has frequently been associated with serotype a in different countries, and belongs to a cluster of related STs (Meats et al. 2003, Ulanova & Tsang 2014). ST-23 and unrelated ST-4 serotype a have been described as those causing meningitis in Brazil (Lima et al. 2010). ST-6 represents the major clonal group of serotype b recovered from patients with invasive disease (Meats et al. 2003). MLST of Brazilian Hib has not been previously published. We identified only three spontaneously occurring capsular deficient mutants of Hib (b^-^), all belonging to cc6. These b^-^ variants have also been recovered from patients with invasive disease at a low frequency in other studies (Satola et al. 2007, Zanella et al. 2011). The non-encapsulated isolates showed more genetic diversity than encapsulated ones and were generally recovered from very young children. Interestingly, two of these isolates shared a *pgi* allele with serotypes b or e, and belonged to STs, which clustered within the clonal complex normally associated with these serotypes (Meats et al. 2003). This raises the possibility that these isolates may be Hib or e that have lost the capsule polysaccharide synthesis genes (Ogilvie et al. 2001).

It has been suggested that the incorporation of the IS*1016-bexA* mutation increased the virulence of serotype a isolates in north-east Brazil (Lima et al. 2010). This mutation was observed in isolates of one of the two PFGE patterns of serotype a, which was ST-4, and was associated with a worse prognosis. In 2007, one ST-4 serotype a isolate with the IS*1016*-*bexA* mutation was recovered from a patient in Canada (Sill et al. 2007). This mutation was also found in serotype a isolates recovered from patients with invasive disease in the US and West Africa (Kroll et al. 1994, Adderson et al. 2001, Kapogiannis et al. 2005). The occurrence of a severe disease resembling meningococcemia has been reported in the presence of the IS*1016*-*bexA* deletion (Adderson et al. 2001). However, invasive Hia isolates have not shown presence of IS*1016*-*bexA* deletion in other studies (Adderson et al. 2001, Hammitt et al. 2005, Tsang et al. 2006). Regardless, the clinical aspects of serotype a disease closely resemble to those of serotype b.

In this study, we did not confirm an association of IS*1016-bexA* deletion with outcome. The IS*1016-bexA* partial deletion was only observed among serotype b isolates, which produced a similar or lower CFR compared with serotype a isolates. Furthermore, serotype a isolates were more often associated with a haemorrhagic rash – a clinical sign of septicaemia and an immediate indicator of prognosis. Together, these data reinforce the view that properties other than IS*1016-bexA* partial deletion may contribute to the development of severe invasive disease, including increased capsular polysaccharide production, variations in gene expression, lipopolysaccharide sialylation, or host factors (Ogilvie et al. 2001, Gilsdorf et al. 2004).

Notably, our CFR was high and increased after 2010, which may not be related to the properties of Hi. Thus, it seems to be necessary to evaluate the independent factors associated with a high CFR. The time lapse after disease onset influences the outcome in patients with bacterial meningitis or septicaemia, making a poor prognosis possibly related to the degree of alertness of the attendant physician, poor access to hospitals, delayed treatment, or limited availability of supportive intensive care.

β-lactam resistance in Hi emerged in the early 1970s and spread globally. The proportion of ampicillin resistance varies by geographical area, with resistance ranging from 3% to 65% (Tristram et al. 2007). It seems that ampicillin resistance is related only to β-lactamase production in the isolates tested, since all β-lactam resistant isolates were susceptible to amoxicillin-clavulanic acid. The β-lactamase-positive isolates studied were resistant to both ampicillin and chloramphenicol, but after 2001, chloramphenicol resistance was not detected. Ampicillin resistance seems to be more commonly found in Hib than in serotype a, as shown in this study (Shuel et al. 2014).

Here, we demonstrated the need to implement an enhanced surveillance system to continue monitoring the impact of Hib conjugate vaccine. Thus, an improvement in typing isolates, full population-based information on incidence, and continued molecular surveillance for invasive Hi disease is necessary, where the disease is still with us and has a great chance to cause the patient’s death.
